# Inhibition of ABCB1 (MDR1) Expression by an siRNA Nanoparticulate Delivery System to Overcome Drug Resistance in Osteosarcoma

**DOI:** 10.1371/journal.pone.0010764

**Published:** 2010-05-24

**Authors:** Michiro Susa, Arun K. Iyer, Keinosuke Ryu, Edwin Choy, Francis J. Hornicek, Henry Mankin, Lara Milane, Mansoor M. Amiji, Zhenfeng Duan

**Affiliations:** 1 Department of Orthopaedic Surgery, Massachusetts General Hospital, Boston, Massachusetts, United States of America; 2 Sarcoma Biology Laboratory, Center for Sarcoma and Connective Tissue Oncology, Massachusetts General Hospital, Boston, Massachusetts, United States of America; 3 Department of Pharmaceutical Sciences, School of Pharmacy, Northeastern University, Boston, Massachusetts, United States of America; Dana-Farber Cancer Institute, United States of America

## Abstract

**Background:**

The use of neo-adjuvant chemotherapy in treating osteosarcoma has improved patients' average 5 year survival rate from 20% to 70% in the past 30 years. However, for patients who progress after chemotherapy, its effectiveness diminishes due to the emergence of multi-drug resistance (MDR) after prolonged therapy.

**Methodology/Principal Findings:**

In order to overcome both the dose-limiting side effects of conventional chemotherapeutic agents and the therapeutic failure resulting from MDR, we designed and evaluated a novel drug delivery system for MDR1 siRNA delivery. Novel biocompatible, lipid-modified dextran-based polymeric nanoparticles were used as the platform for MDR1 siRNA delivery; and the efficacy of combination therapy with this system was evaluated. In this study, multi-drug resistant osteosarcoma cell lines (KHOS_R2_ and U-2OS_R2_) were treated with the MDR1 siRNA nanocarriers and MDR1 protein (P-gp) expression, drug retention, and immunofluoresence were analyzed. Combination therapy of the MDR1 siRNA loaded nanocarriers with increasing concentrations of doxorubicin was also analyzed. We observed that MDR1 siRNA loaded dextran nanoparticles efficiently suppresses P-gp expression in the drug resistant osteosarcoma cell lines. The results also demonstrated that this approach may be capable of reversing drug resistance by increasing the amount of drug accumulation in MDR cell lines.

**Conclusions/Significance:**

Lipid-modified dextran-based polymeric nanoparticles are a promising platform for siRNA delivery. Nanocarriers loaded with MDR1 siRNA are a potential treatment strategy for reversing MDR in osteosarcoma.

## Introduction

Drug resistance to chemotherapeutic agents is one of the major obstacles in the treatment of human cancers. Cancer cells employ a host of different mechanisms to become resistant to one or more chemotherapeutic agents. One of the major causes of multidrug resistance (MDR) is the overexpression of membrane bound drug transporter proteins, such as P-glycoprotein (P-gp, ABCB1), multidrug resistance-associated proteins (MRP1, ABCC1 and MRP2, ABCC2), and breast cancer resistance protein (BCRP, ABCG2) [1].

Osteosarcoma accounts for approximately 60% of primary malignant bone tumors diagnosed in the first two decades of life [2]. The cure rate for patients with localized osteosarcoma ranges from 15% to 20% with surgery alone, but improves dramatically to approximately 70% when combined with chemotherapy [3]. Unfortunately, the efficacy of chemotherapy is hampered by the eventual development of MDR. It is estimated that less than 30% of patients with recurrent disease will be cured [3], [4], [5].

The development and discovery of agents that reverse MDR with high efficiency and low toxicity is the focus of extensive research [6], [7], [8], [9]. Unfortunately, these compounds are often nonspecific and have low efficiency and/or high toxicity; as such, phase 3 clinical trials of these agents are largely disappointing [6], [7], [10], [11], [12], [13]. Consequently, it is imperative to develop alternative, less toxic and more efficient strategies to overcome MDR.

One of the innovative approaches to addressing MDR is to inhibit MDR1 mRNA expression by RNA interference (RNAi). RNAi is a technique that mimics and exploits endogenous silencing mechanisms resulting in post-transcriptional gene silencing of double-stranded RNA inside cells. The double-stranded 21 to 23 nucleotide noncoding small interfering RNA (siRNA) can knockdown expression of genes in a highly efficient and sequence specific manner. The efficiency of RNAi and its limited side effects have made this technique an attractive alternative to the use of antisense oligonucleotides and ribozymes for therapies based on the inhibition of target genes [14]. Previously, we have demonstrated that both synthetic-based and plasmid-based siRNA can significantly block MDR1 expression in drug resistant cell lines [15]. Although the RNAi technology is an excellent candidate for cancer therapy, several challenges need to be addressed for clinical application. First, the poor membrane permeability of siRNA limits cellular uptake. Secondly, most of the reagents for delivery of siRNA such as Lipofectamine™ are toxic. Third, siRNA is unstable and rapidly degraded by nucleases. Further use of siRNA as therapeutic agents will rely mostly on the development of more efficient delivery systems.

Nanotechnology offers solutions to overcome the adversity of siRNA delivery. Several varieties of nanoparticles are available including polymeric nanoparticles, dendrimers, inorganic/metal nanoparticles, quantum dots, liposomes, and micelles [16]. The versatility of polymeric nanocarriers offers a significant advantage over other nano-carrier platforms; polymer matrices can be selected according to utility which allows for the customization of nanoparticle properties. Additional advantages of polymeric nanocarriers include ease in surface modification, greater encapsulation efficiency of the payload, payload protection, large surface area-to-volume ratio, and the ability to modify the rate of polymer erosion for temporal control over the release of nucleotides [17]. Among the natural polysaccharides, dextran based nanoparticles have been used to deliver chemotherapeutic drugs which resulted in enhancement of drug accumulation and increased apoptosis in cancer cells [18] with limited cytotoxicity (Fig. S1). Dextran has been widely studied for utilization as polymeric drug carrier due to its biocompatibility and biodegradability [19]. In a recent report, Zhang et al. used biocompatible and non-toxic pH sensitive dextran nanoparticles for drug delivery application [20]. In this report, we analyzed the effect and stability of MDR1 siRNA loaded dextran nanoparticles on multidrug resistant osteosarcoma cell lines.

## Materials and Methods

### Chemicals

Dextran (Mw∼40kDa), stearyl amine (99% pure), cystamine, pyridine, sodium periodate (NaIO_4_), sodium cyanoborohydride (NaCNBH_3_), potassium sulfate (K_2_SO_4)_ and azo-bis-isobutyronitrile (AIBN) were obtained from Sigma-Aldrich Chemical Co (St. Louis, MO). Dithiol-modified poly(ethylene glycol) (PEG-(SH)_2_, M.W. 2,000) was purchased from SunBio, Inc. (Seoul, South Korea). Anhydrous lithium chloride (LiCl) was obtained from Fisher Scientific (Philadelphia, PA). Dehydrated dimethylformamide (DMF) and dimethylsulfoxide (DMSO) with molecular sieves were obtained from Acros Organics (Parsipanny, NJ). Acryloyl chloride, pyridine and other reagents and solvents were from Sigma-Aldrich and were used as received without further purification.

### Synthesis of ABCB1 siRNA

The siRNA sequence targeting ABCB1 (Genbank accession no. NM_000927) corresponded to coding regions of these genes. Four target sequences were selected for the ABCB1 gene. Sense sequences for each siRNA were 5′ GAGCUUAACA CC CGA CUUAUU 3′, 5′ GAAAGUAUACCUCCAGUUUUU 3′, 5′ GAC CAUAAAU GUAAG GUUUUU 3′, and 5′ CCAGGUAUGCCUAUUAUUAUU 3′. Synthetic siRNA duplexes were obtained from Dharmacon Inc. (Layfayette, CO). The siRNAs were dissolved by adding 1 mL of the buffer [100 mmol/L potassium acetate, 30 mmol/L HEPES-KOH, and 2 mmol/L magnesium acetate (pH 7.4)] to each tube, and stored at −20°C until the following transfection experiment. Non-specific siRNA which has no significant homology to any known gene sequences from mouse, rat, or human was obtained from Ambion (Austin, Tx).

### Synthesis of lipid-modified dextran polymer

#### Synthesis of dextran acrylate

The synthesis of dextran acrylate was based on the procedure of Zhang et al. [21]. Briefly, a fixed amount of dextran (M.W. ∼40kDa, 2 g) was added to a LiCl/DMF (4% w/v, 50 ml) solvent mixture in a round bottom flask (200 ml). The temperature of the oil bath was raised from room temperature to 120°C over a period of 2 h. The resultant mixture became a homogeneous golden yellow colored solution. The solution was cooled to room temperature, and pyridine (500 µl) was added and stirred. The reaction mixture was cooled to 0°C using ice bath and varying amounts of acryloyl chloride (1–1.5 molar excess) were added drop wise using an addition funnel. The reaction was maintained at 0°C until complete addition of acryloyl chloride was done over a period of 1–2 h. The reaction was allowed to continue overnight. The dextran-acrylate obtained was precipitated in excess cold ethanol and washed three times with absolute ethanol. For confirmation of the formation of dextran acrylate, a small portion of the acrylate monomer was polymerized using 0.001% AIBN initiator in DMSO at 60°C for 24 hours, which resulted in formation of the acrylate polymer, confirming the reaction. Alternately, for lipid modification, the dextran-acrylate obtained in step 1 was directly used as the monomer for the next step, in a one-pot synthesis.

#### Lipid modification of dextran acrylate

**Figure 1:**
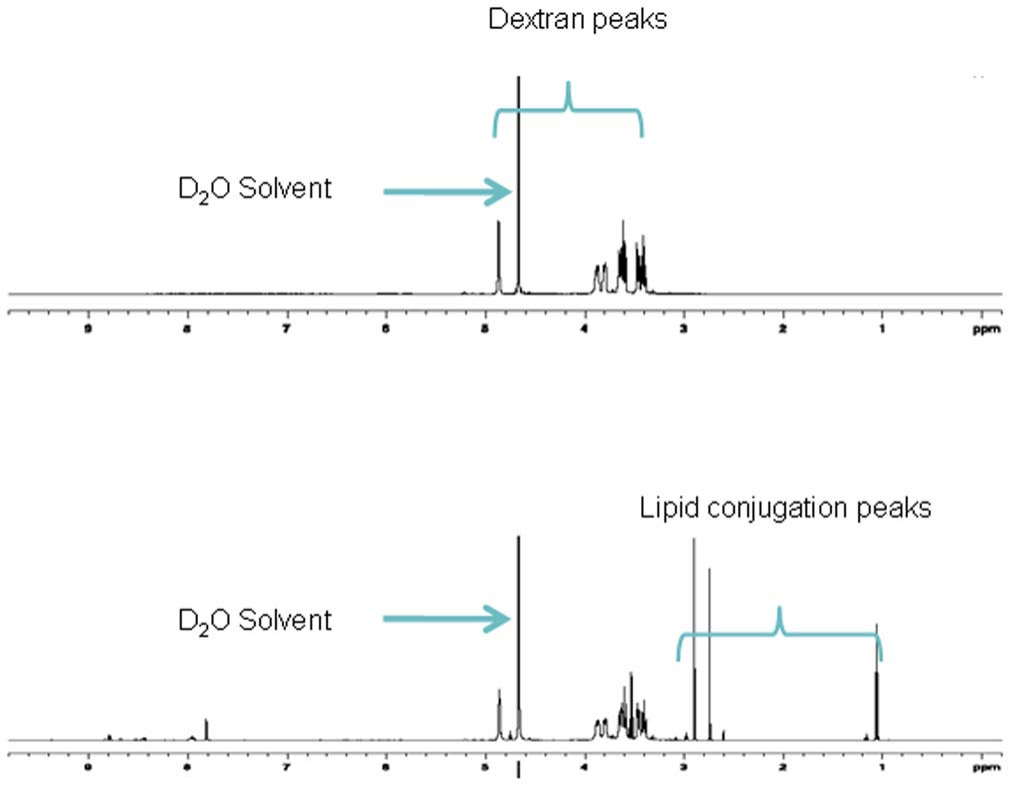
Characterization of stearylamine-dextran modified nanoparticles. 500 MHz ^1^H NMR spectra of dextran and dextran conjugated stearyl amine after purification using D_2_O as the solvent. The spectra clearly show the additional peaks of the long chain fatty amine at ∼1ppm indicating the successful lipid modification of dextran. The % fat modification was estimated to be 7 mole % of dextran.

A desired amount (200mg) of dextran acrylate obtained from above step was dissolved in dry DMF and stirred in a 20ml glass vial with varying amounts (5–10 mole %) of stearylamine and a catalyst (0.01 mole % AlCl_3_). The reaction mixture was heated to 40–50°C in an oil bath for 24 h. The product obtained (stearylamine - modified dextran) was precipitated and washed in cold ethanol several times to purify the product. Finally, the lipid-modified dextran derivative was dissolved in small amount of deionized water and lyophilized to yield the pale yellow colored final product. The fatty amine modification of dextran was confirmed by ^1^H NMR spectroscopy (Varian 500MHz NMR spectrometer, Varian Inc, CA) and the % lipid modification was estimated to be 7 mole% (Fig. 1).

### Synthesis of thiolated dextran

#### Oxidation of dextran

Dextran is an α-D-1, 6-glucose-linked glucan with side chains 1–3 linked to the backbone units of the dextran biopolymer. Dextran was selected for the development of the macrostructure based on its long history of use as plasma expander and drug delivery platform. The dextran backbone was oxidized based on the procedure of Martwiset et al. [22]. Briefly, a desired amount of NaIO_4_ was dissolved in 60 ml of deionised water. The solution was added to another solution containing 4 g of dextran and 30 ml of de-ionised water. The reaction was stirred in the dark for 2 h at room temperature. At the end of the reaction, the solution was dialyzed using Spectrapor® dialysis membranes (M.W. cutoff 12–14 kDa, Spectrum Labs, Rancho Dominguez, CA) against de-ionized water (2L) for 4 days with several water replacements. A powdery free-flowing sample was obtained after freeze-drying. Yield: 3.7 g (92.5%).

#### Thiol modification of dextran

A 500 mg portion of the oxidized dextran was dissolved in 50 ml pH 5.2 buffer containing K_2_SO_4_ and NaCNBH_3_. Then 50 mg of cystamine was added and stirred at 40°C for 4 days. The product was subjected to extensive dialysis and then lyophilized to yield thiolated-dextran. The percent thiolation was quantified by Ellman's reagent [23]. The concentration of sulfohydryl groups in the purified thiolated dextran derivate was estimated to be ∼14.2 µmole/mg.

### Preparation of MDR1 siRNA-containing dextran nanoparticles

The MDR1 siRNA containing dextran nanoparticles were prepared in a similar fashion as reported recently for the preparation of doxorubicin loaded dextran nanosystems [18]. A stock solution of 5 mg/ml (∼2.5 mM) dextran thiol, 5 mg/ml (∼2.5 mM) dextran stearylamine, 5 mg/ml (125 µM) PEG-thiol, and 2 µM MDR1 siRNA was prepared. For obtaining a 100 nM siRNA concentration, 120 µl stock solution of MDR1 siRNA was mixed with 40 µl of dextran-thiol stock solution using a vortex shaker. It was then incubated for 5 minutes. To this mixture, 40 µl dextran-stearylamine derivative was then added and incubated for another 5 mins. Finally 40 µl of PEG-(SH)_2_ was added and incubated for another 15 minutes to form the hydrophilic shell of the nanoparticles. This method of sequential addition was used so that the siRNA is entrapped in the interpenetrating (IPN) dextran hydrogel network. Further, a PEG-thiol derivative was used to provide stealth character to the nanoparticles. The final mixture was subsequently diluted with the medium and applied at the designated concentrations. Nanoparticles containing 100 nM non-specific siRNA was used as negative controls in some experiments.

### Particle size and zeta potential measurements

The particle size and zeta potentials of MDR1 siRNA loaded nanoparticles were performed with a Brookhaven Zeta PALS Instrument (Holtsville, NY). For light scattering experiments, the samples were measured at fixed angle of 90° at 25°C. The scattering intensity was adjusted in the range of 50–500 kcps by diluting the samples with deionised water. For zeta potentials, default parameters of dielectric constant, refractive index, and viscosity of water were used based on the electrophoretic mobility of the nanoparticles.

### Cell culture and reagents

Dr. Efstathios S. Gonos (National Hellenic research Foundation, Athens, Greece) kindly provided the human osteosarcoma cell line KHOS and the multidrug resistant MDR1 (P-gp) expressing cell lines KHOS_R2_ and U-2OS_R2_. All cell lines were cultured in RPMI 1640 medium supplemented with 10% fetal bovine serum, 100 U/ml penicillin, and 100 ug/ml streptomycin (all obtained from invitrogen, Carlsbad, CA). Cells were incubated at 37°C in 5% CO2-95% air atmosphere and passaged when near confluent monolayers were achieved using trypsin-EDTA solution. Resistant cell lines were continuously cultured in 0.1uM doxorubicin. Doxorubicin was obtained as unused residual clinical material at the Massachusetts General Hospital. The P-gp1 monoclonal antibody C219 was purchased from Signet (Dedham, MA). The human β-actin monoclonal antibody and the MTT reagents were purchased from Sigma-Aldrich (St. Louis, MO).

### Generation of EGFP expressing cells and transfection by EGFP siRNA loaded nanoparticles

The Stat3 and enhanced green fluorescent protein (EGFP) fusion protein expression vector pCORON1000 EGFP-Stat3 (abbreviated as pEGFP-Stat3) was purchased from Amersham Biosciences (Buckinghamshire, United Kingdom). This pEGFP-Stat3 vector was generated by fusing Stat3 to the COOH terminus of EGFP. A hamster kidney cell line (BHK-21) was stably transfected with pEGFP-Stat3 through selection with G418 (Invitrogen, Carlsbad, CA). EGFP expressing cells were seeded at a density of 4000 cells per well in 96-well plates with addition of increased concentration of EGFP siRNA and subsequently incubated for 48 hr. EGFP siRNA was purchased from Ambion (Austin, Tx). siPORT™ NeoFX™Transfection Agent was used as a positive control (Ambion). Images were acquired by Nikon Eclipse Ti-U fluorescence microscope (Nikon Corp.) equipped with a SPOT RT digital camera (Diagnostic Instruments, Inc., Sterling Heights, MI).

### Western Blot analysis

P-gp1 was analyzed in total cell lysates. Protein lysates from cells were generated through lysis with 1× RIPA Lysis Buffer (Upstate Biotechnology, Charlottesville, VA). The concentration of the protein was determined by Protein Assay reagents (Bio-Rad, Hercules, CA) and a spectrophotometer (Beckman DU-640, Beckman Instruments, Inc., Columbia, MD). 25 µg of total protein was processed on Nu-Page 4–12% Bis-Tris Gel (Invitrogen) and transferred to a pure nitrocellulose membrane (Bio-Rad Laboratories, Hecules, CA). Primary antibodies were incubated in Tris-buffered saline, pH 7.4, with 0.1% Tween 20 overnight at 4°C. Signal was generated through incubation with horseradish peroxidase-conjugated secondary antibodies (Bio-Rad, Hercules, CA) incubated in Tris-buffered saline, pH 7.4, with 5% nonfat milk and 0.1% Tween 20 at 1 ∶ 2000 dilution for 1h at room temperature. Positive immunoreactions were detected by using Super Signal ®West Pico Chemiluminescent Substrate (Pierce, Rockford, IL).

Bands were semi-quantified by reverse image scanning densitometry with PhotoShop 7.0 (Adobe, San Jose, CA). An area of the gel image that was devoid of signal was assigned to be the background value. Then each band of the protein representing P-gp from KHOS_R2_ or U2OS_R2_ treated with various concentrations of MDR1 siRNA nanoparticles was analyzed for density beyond the background level. To ensure that the loading of the protein was equal and differences were not being observed because of one specimen having more protein than another, the band corresponding to β-actin was determined for each protein. The density of the protein band was normalized to the β-actin band for the protein and the ratio of the P-gp was normalized by dividing by the ratio of the β-actin corresponding to each P-gp.

### Duration of MDR1 reversal

For comparison of the duration of MDR1 inhibition by either MDR1 siRNA with siPORT™ NeoFX™ Transfection Agent or MDR1 siRNA loaded nanoparticles, 1×10^5^ KHOS_R2_ cells/well were incubated with each MDR1 siRNA combinations for 5 days. The expression of P-gp was determined by western blot analysis as described above.

### Drug Efflux Assay

The Vybrant™ multi-drug resistance assay kit (Invitrogen/Molecular Probes) was used to measure the drug efflux properties of the resistant cell lines. This assay utilizes the fluorogenic dye calcein acetoxymethyl ester (calcein AM) as a substrate for efflux activity of P-gp or other membrane pump ABC proteins. Calcein AM is taken up by cells and hydrolyzed by cytoplasmic esterases into fluorescent calcein. Calcein is well retained in the cytosol. However, multidrug resistant cells expressing high levels of P-gp rapidly extrude non-fluorescent calcein AM from the plasma membrane, reducing accumulation of fluorescent calcein in the cytosol. Drug resistant cells (1×10^5^) were cultured in 96-well plates with either increasing concentrations of MDR1 siRNA loaded nanoparticles or with media alone. After 48 hours, they were incubated with 0.25 µM calcein AM in 150 µl total volume. Ten µM Verapamil was used as a positive control. PBS and nanoparticles loaded with 100 nM nonspecific siRNA were used as negative controls. After 30 minutes, the cells were washed and centrifuged twice with 200 µl cold RPMI1640 culture medium, and cell fluorescence was measured at a wavelength of 490 nm (A_490_) on a SPECTRAmax® Microplate Spectrofluorometer (Molecular Devices). For visualization of the intracellular retention of calcein AM, images were acquired by Nikon Eclipse Ti-U fluorescence microscope (Nikon Corp.) equipped with a SPOT RT digital camera (Diagnostic Instruments, Inc., Sterling Heights, MI).

### Fluorescence microscopy of cellular doxorubicin uptake

For cellular uptake studies, KHOS and KHOS_R2_ cells were seeded at densities of 5×10^5^ cells/well in 6 well plates. MDR1 siRNA was applied to a well of KHOS_R2_ and incubated for 48 h. Following the incubation, doxorubicin was added to each well and was incubated for additional 3 hours. After incubation, the cells were washed, suspended in fresh RPMI 1640, then visualized on a Nikon Eclipse Ti-U fluorescence microscope (Nikon Corp.) equipped with a SPOT RT digital camera (Diagnostic Instruments, Inc., Sterling Heights, MI). Fluorescence intensity and cellular localization was analyzed at a wavelength of 488 nm in triplicate in random, different fields.

### *In vitro* Cytotoxicity assay

In vitro cytotoxity assays were performed by MTT assay as previously described. Briefly, 3×10 ^3^ cells per well were plated in a 96-well plate during this process. After 48 hours of incubation with MDR1 siRNA loaded nanocarriers, nonspecific siRNA loaded nanocarriers or with medium alone, increasing concentrations of doxorubicin were applied. After culturing for 5 days, 10 µl of MTT (5 mg/ml in PBS) was added to each well and incubated for 3 hr. After dissolving the resulting formazan product with acid isopropnol, the absorbance (A490) was read on a SPECTRA max Microplate Spectrophotometer (Molecular Devices) at a wavelength of 490 nm. Each experiment was performed in triplicate.

### Statistical analysis

Student's t-test was used to compare the differences between groups (GraphPad PRISM® 4 software, GraphPad Software, San Diego, CA). Results are given as mean ±SD and results with p<0.05 were considered statistically significant.

## Results

### Lipid-modified dextran nanoparticles for intracellular MDR1 siRNA delivery

We have recently developed a self assembled nanosystem based on dextran nanoparticles to stably encapsulate doxorubicin [18]. We used a similar sequential addition method with some modifications to encapsulate MDR1 siRNA. The seqential addition method resulted in high encapsulation efficiency of >95% into the nanoparticles as observed from a FITC-labeled scrambled sequence siRNA encapslation and release study (data not shown). The mean particle size of the MDR1 siRNA loaded nanoparticles as determined by dynamic light scattering (DLS) measurement was 104.4±3.7 nm and the zeta potential was almost neutral (−0.19±1.13 mV), indicative of the entrapment of the siRNA within the interpenetrating dextran hydrogel network. Further, the particles were stable at room temperature or at 4°C and there was minimal change in the particle size upon storage (for 1 week at 4°C) as determined by DLS measurements.

### Effect of EGFP siRNA loaded nanoparticle on BHK-21-EGFP cells

**Figure 2:**
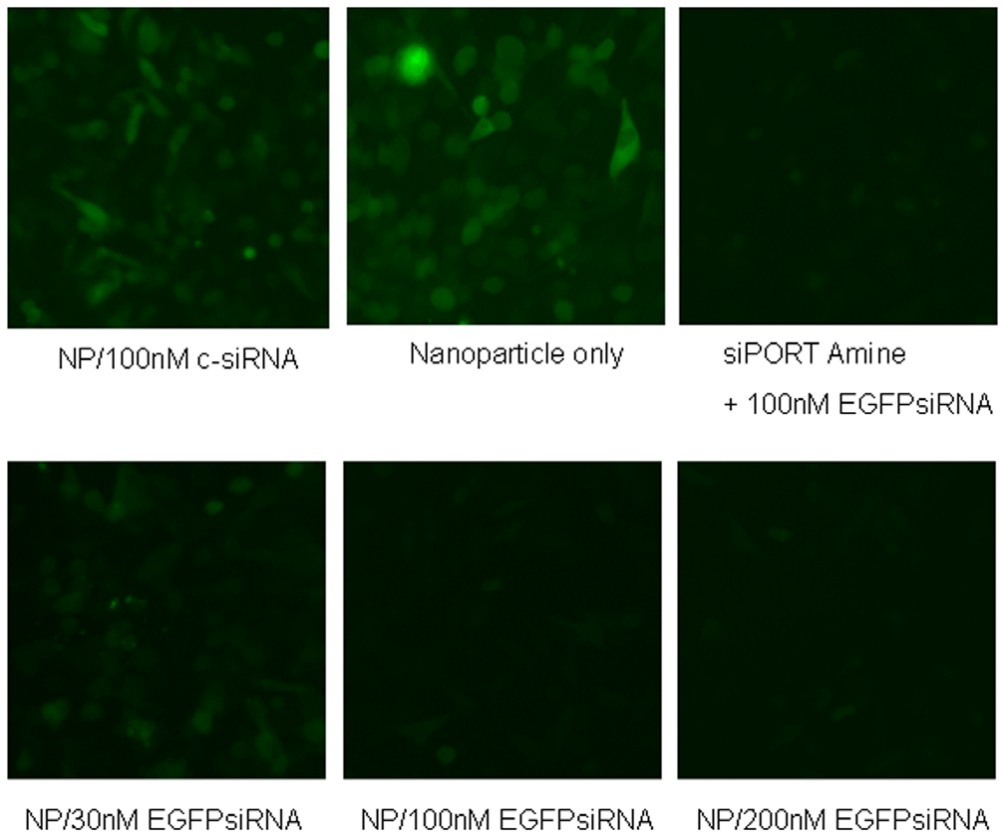
Effect of EGFP siRNA loaded nanoparticles on BHK-21-EGFP cells was assessed. EGFP siRNA was efficiently incorporated into cells and effectively inhibited the expression of EGFP in a dose dependent manner (NP: nanoparticle). siPORT™ NeoFX™ Transfection Agent (Ambion) was used as a positive control. Nanoparticles loaded with 100 nM non-specific siRNA (c-siRNA) and unloaded nanoparticles were used as negative controls.

To assess the transfection efficacy of siRNA loaded nanoparticles on cell lines, we first utilized EGFP expressing BHK-21 cells to test the effects. The EGFP siRNA loaded nanoparticles were non-toxic at the concentrations utilized in this study (Fig. S2). The EGFP siRNA was efficiently incorporated into cells and effectively inhibited the expression of EGFP in a dose dependent manner, but reached a plateau at approximately 100 nM (Fig. 2).

### Stable suppression of P-gp using MDR1siRNA loaded nanoparticle

**Figure 3:**
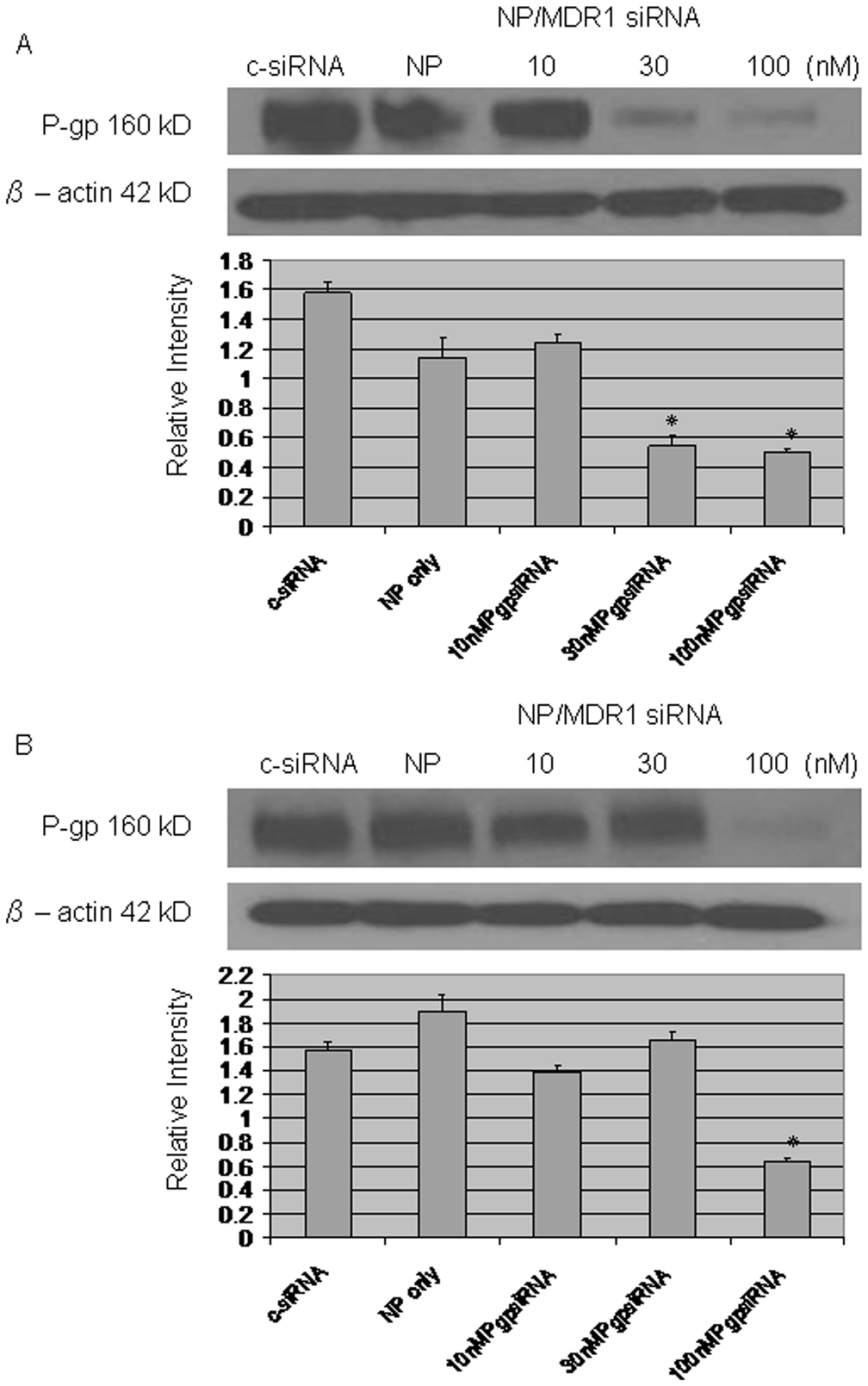
Western blot assay was performed to assess the effect of MDR1 siRNA loaded nanoparticles on expression of P-gp. The expression of P-gp was significantly decreased after application of 30 nM MDR1 siRNA loaded nanoparticles for KHOS_R2_ (*A*) and 100 nM MDR1 siRNA loaded nanoparticles for U-2OS_R2_ (*B*). *P* values are shown as follows: *P<0.05.

**Figure 4:**
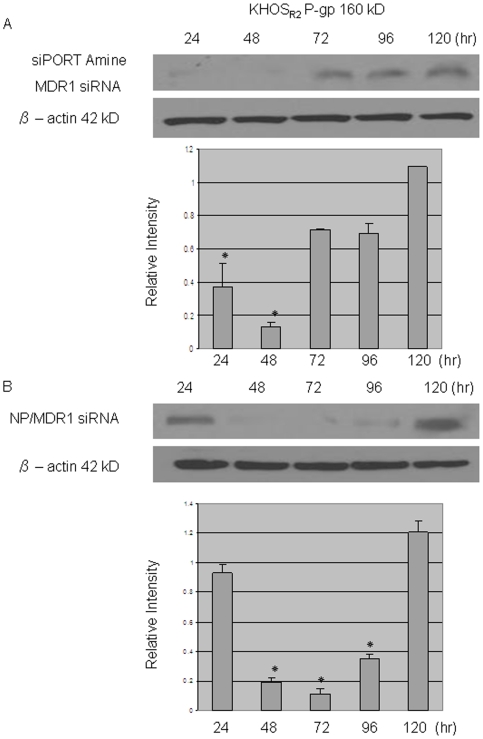
Western blot assay was performed to assess the duration of MDR1 siRNA loaded nanoparticle. siRNA transfected with siPORT™ NeoFX™ Transfection Agent was able to suppress P-gp expression for 48 hours (*A*). Inhibition of P-gp started slower when MDR1 siRNA was loaded in to nanoparticles, but exhibited longer inhibition compared to MDR1 siRNA transfected with commercially available agent (*B*). *P* values are shown as follows: *P<0.05.

Western blotting was performed to estimate the effect of MDR1 siRNA loaded nanoparticle on P-gp expression. P-gp expression has been previously confirmed in the two drug resistant cell lines KHOS_R2_ and U-2OS_R2_ (data not shown). MDR1 siRNA loaded nanoparticle inhibited the expression of P-gp at a concentration of as low as 30 nM (Fig. 3A, B). siRNA transfected with siPORT™ NeoFX™ Transfection Agent was able to suppress P-gp expression for 48 hours (Fig. 4A). siRNA loaded nanoparticles were slower in achieving the suppression of P-gp, but were able to maintain suppression for 96 hours (Fig. 4B).

### MDR1 siRNA loaded nanoparticle inhibits P-gp-mediated efflux of calcein AM

**Figure 5:**
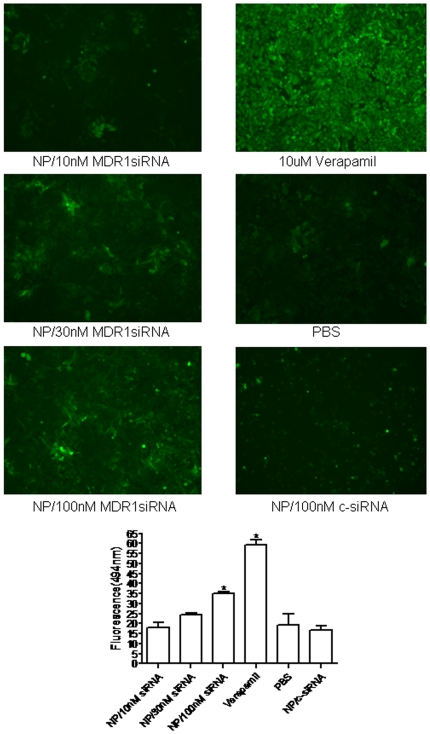
Effect of MDR1 siRNA loaded nanoparticle on P-gp – mediated uptake and efflux was analyzed using calcein AM. Cells treated with MDR1 siRNA were shown to decrease calcein AM efflux in a dose-dependent manner as determined by image analysis, and this was confirmed by microplate spectrofluorometer analysis. *P* values are shown as follows: *P<0.05.

Reversal of MDR is usually manifested as an increased intracellular accumulation of chemotherapeutics, which can be achieved by disturbing P-gp-mediated drug uptake and efflux. Therefore, the effect of MDR1 siRNA loaded nanoparticles was examined for the uptake and efflux of a P-gp substrate, calcein AM, in KHOS_R2._ Cells treated with MDR1 siRNA were shown to decrease calcein AM efflux in a dose-dependent manner as determined by image analysis, and confirmed by microplate spectrofluorometer analysis (Fig. 5).

### Enhancement of intracellular doxorubicin accumulation with MDR1 siRNA loaded nanoparticle delivery

**Figure 6:**
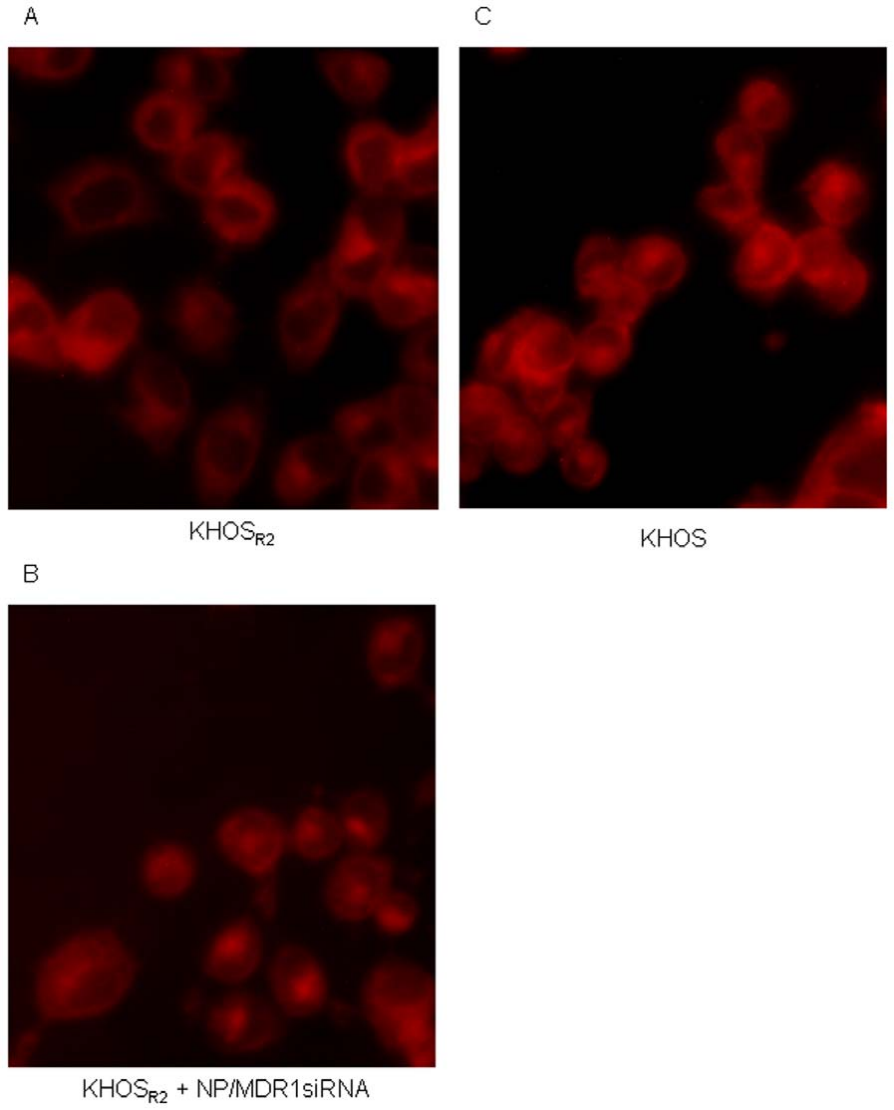
Subcellular distribution of doxorubicin in drug sensitive and resistant osteosarcoma cell lines was analyzed under fluorescence microscope (*A-C*). A prominent increase in fluorescence was observed in the nucleus when multidrug resistant cells were treated with doxorubicin after treatment with MDR1 siRNA loaded nanoparticles.

Using fluorescent microscopy, subcellular distribution of doxorubicin in KHOS and KHOS_R2_ was analyzed. After a 3 hour incubation period with free doxorubicin in drug resistant osteosarcoma cells, the drug was primarily concentrated in the cytoplasm with a very low level of fluorescence observed in the nucleus (Fig. 6A). When doxorubicin was administered after treatment with MDR1 siRNA loaded nanoparticle to drug resistant cell lines, an increase in fluorescence was observed in the nucleus and cytoplasm (Fig. 6B). This subcellular distribution mimicked that of the drug sensitive variant when treated with doxorubicin (Fig. 6C).

### *In vitro* cytotoxicity studies in drug resistant osteosarcoma cells

**Figure 7:**
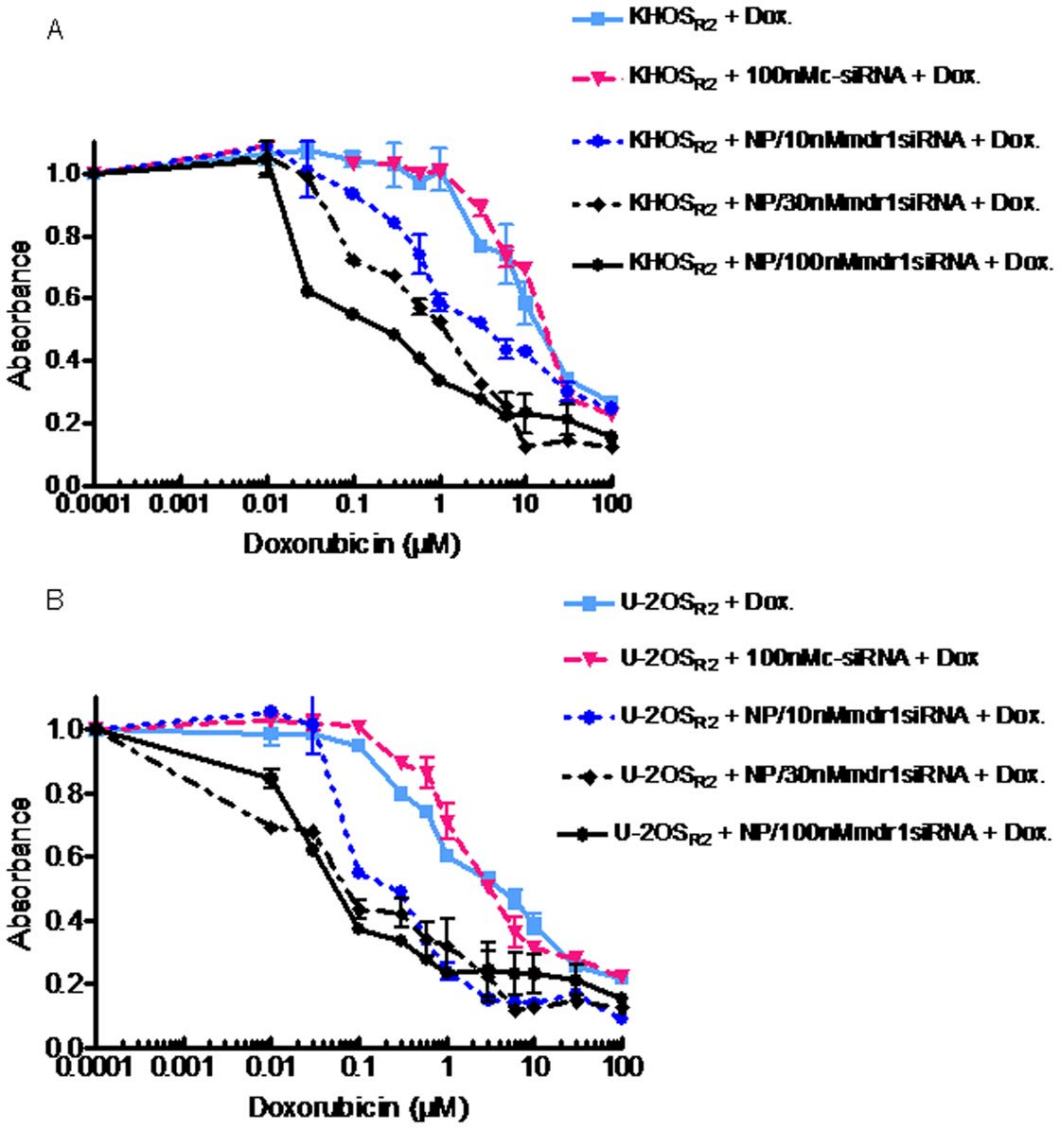
The effect of doxorubicin alone or nanoparticle loaded with MDR1 siRNA on KHOS_R2_ and U-2OS_R2_ was analyzed. Varying concentrations of doxorubicin was added and cells were cultured for 5 days. Treatment with nanoparticles loaded with MDR1 siRNA showed increased anti-proliferative activity in both drug resistant osteosarcoma cell lines, KHOS_R2_ (*A*) and U-2OS_R2_ (*B*), in a dose dependent manner. Growth inhibition was assessed by MTT as described under Materials and Methods. The experiment was repeated four times in triplicate.

The dextran nanoparticles are not cytotoxic by themselves at the dose used in this study (data not shown). After treatment with MDR1 siRNA loaded nanoparticles, we found that doxorubicin showed an increased amount of anti-proliferative activity in drug resistant osteosarcoma cell lines in a dose dependent manner (Fig. 7A, B). With the delivery of nanoparticles loaded with 100 nM MDR1 siRNA, growth inhibition with doxorubicin was substantially more marked than with the administration of 100 fold higher amounts of free drugs. For example, in the KHOS_R2_ cell line the IC_50_ of doxorubicin alone was 10 µM which was reduced to 0.1 µM when the cells were co-treated with MDR1 siRNA loaded nanoparticles. Likewise, the drug resistant cell line U-2OS_R2_ displayed a similar trend; the IC_50_ for doxorubicin alone was 6 µM which was reduced to 0.06 µM when co-treated with the MDR1 siRNA loaded nanoparticles.

## Discussion

The primary limitation for the successful treatment of osteosarcoma is the development of MDR against various chemotherapeutic agents. Adaptation of cells to drug exposure involves activation of drug efflux pumps, increased cellular detoxification, and up-regulation of repair mechanisms, all of which lead to the development of multidrug resistant character in cancer cells [24]. Overexpression of the ATP binding cassette transporters such as ABCB1 (MDR1) has been directly implicated in resistance to a broad spectrum of chemotherapeutic agents in vitro including anthracyclines, paclitaxel, and the Vinca alkaloids [25], [26], [27]. For several types of cancer, ABCB1 overexpression may be the predominant factor in limiting the efficacy of chemotherapeutic agents. Overexpression of the ABCB1 gene within drug-sensitive cells or mice to produce transgenic animals confers resistance to the agents described [28], [29].

The objective of this study was to investigate the ability of MDR1 siRNA loaded dextran based nanoparticles to overcome P-gp mediated drug resistance in osteosarcoma. We developed a biocompatible and safe nanoparticulate system for the delivery of MDR1 siRNA, similar to the design reported recently by us for self-assembled nanosystems for encapsulating doxorubicin [18]. For this purpose, we first incubated the MDR1 siRNA with a thiol-modified dextran derivative to entrap the siRNA in the dextran network. This was followed by a sequential addition of a stearylamine modified dextran derivative (to form an interpenetrating dextran hydrogel network) and a thiolated PEG derivative to stabilize the nanoparticles. The combination of several dextran-lipid derivatives and dextran-thiol polymers and their concentrations were arrived at after several testing of the formulation for their ability to form stable nanoparticles as assessed by dynamic light scattering. Also the cytotoxicity testing of various dextran derivatives were performed to assess their safe use (Fig S1). This selection approach resulted in the development of a method for formation of stable nanoparticles with good siRNA loading/entrapment, supported by the dynamic light scattering data on the size and charge of the nanoparticles.

We used PEG in our formulation to prepare the nanoparticles because it is well known that PEGylated liposomes and nanoparticles can impart stealth characteristics to the nanoparticles, increasing the plasma residence time, and protecting the drug payload from degradation during circulation [30]. PEGlyated nanoparticles also have the ability to passively target tumor tissues in vivo by exploiting the inherent abnormalities of the leaky tumor vasculature [31]. Further, tumor tissues have impaired lymphatic drainage/recovery system and greatly increased production of a number of vascular permeability mediators [32]. This phenomenon is called the enhanced permeability and retention (EPR) effect [33]. It has been reported that accumulation of nanoparticle bound drugs are 45–250 times higher in the site of tumors compared to other vital organs such as the liver, kidney, lung, spleen, or heart [34].

As a next step for development of nanoparticle delivery system for overcoming drug resistant osteosarcoma, we tested our formulation for siRNA silencing effects in vitro. Our results showed that by treating drug resistant osteosarcoma cell lines with MDR1 siRNA nanoparticles at concentrations of 30 nM or higher, the expression of P-gp was effectively suppressed in both the KHOS_R2_ and the U-2OS_R2_ cells. In addition, MDR1 siRNA loaded nanoparticles were able to suppress the expression of P-gp for a longer period of time compared to MDR1 siRNA transfected with commercially available agents (96 hr compared to 48 hr).

Another major obstacle when inhibiting P-gp is that it is not only present in malignant cells but is also an important constituent of various normal tissues such as peripheral blood cells and hemopoietic progenitors found in normal human bone marrow and the blood brain barrier [35], [36],[37],[38],[39]. In these physiologically normal tissues, P-gp is important in the transport of steroids, the efflux of toxic molecules, the production of bile, and is an important component of cellular defense and protection [40], [41]. Although P-gp expression in these tissues is relatively low, it may play an important role in protecting rapidly dividing cells from toxicity after exposure to anticancer drugs [42]. Consequently, treatment related morbidity and mortality and increased marrow toxicity associated with chemotherapeutics and biologicals that target P-gp limit the clinical application of these agents. To optimize the application of these agents, it is clear that a delivery system that achieves higher specificity for target tissue than use of the agents alone is necessary. Such a system can improve the therapeutic index of siRNA, increasing the potential for clinical application.

Nevertheless, measuring a reduction in total cellular P-gp content does not indicate that the MDR phenotype has been reversed as only the cell surface-bound portion of P-gp is responsible for drug efflux [43]. To confirm the reversal of the MDR phenotype, the Vybrant™ multidrug resistance assay was performed. KHOS_R2_ cells treated with MDR1 siRNA loaded nanoparticles showed increased accumulation of the P-gp substrate calcein-AM by enhancing the uptake and/or decreasing the efflux of these compounds. There is a possibility that off-target effects of the mixed siRNAs used in this study might had some effect on our results, and further investigations using other sequences of MDR1 siRNA is currently underway to confirm the functional assay results. The actual accumulation inside cells, preservation of drug antitumor activity/stability, and subcellular trafficking are important determinants of the efficacy of an anticancer agent. For anthracyclines in MDR cells, it has been reported that in addition to a decrease in drug accumulation, the intracellular distribution of the drug is rearranged in human myeloma [44], myeloid [45], lung tumor cells [46], ovarian carcinoma cells [47], and epidermoid carcinoma cells [48]. Anthracycline fluorescence is found mainly in the nucleus of sensitive cells and in the cytoplasm of cells with relatively high levels of resistance [44], [45], [46], [48]. The question then is whether distribution of doxorubicin will change when drug resistant cells are treated with MDR1 siRNA loaded nanoparticles. Using immunofluorescense microscopy, doxorubicin primarily accumulated in the nucleus of drug sensitive cell lines, and in the cytoplasm of MDR cell lines. When doxorubicin was applied to MDR osteosarcoma cells treated with MDR1 siRNA loaded nanoparticles, drug distribution mimicked that of sensitive cells. The observations indicate that co-treatment with the MDR1 siRNA loaded nanoparticle formulation, by inhibiting P-gp, leads to higher intracellular doxorubicin concentration which allowed drugs to accumulate in the nucleus of MDR cells, emulating the behavior of doxorubicin treatment alone in drug sensitive cells.

Finally, in the treatment of osteosarcoma patients, dose limitation poses significant problems. After treatment with nanoparticles loaded with low concentrations of MDR1 siRNA, drug resistant osteosarcoma cell lines are re-sensitized to doxorubicin as demonstrated by the results of MTT assay and by a higher intracellular accumulation of the drug in the nucleus; demonstrating an uptake and distribution pattern that is comparable to drug sensitive cells. This evaluation suggests that low dosages of doxorubicin/MDR1 siRNA therapy show promise in reducing the systemic lethal side effects which are frequently encountered in the clinical setting.

In conclusion, this report demonstrates the feasibility of using MDR1 siRNA loaded dextran nanoparticles to specifically and effectively modulate MDR. Dextran nanoparticles may be a successful platform to overcome the current delivery limitation of siRNA for the treatment of osteosarcoma.

## Supporting Information

Figure S1 Cytotoxicity of dextran derivatives vs polyethyleneimine(PEI) on NIH3T3 fibroblast (A) and SKOV3 ovarican cancer (B) cells. Varying concentrations of each nanoparticles were added and cells were cultured for 5 days. The mixture of dextran derivatives (thiol+lipid) was found to be almost non-toxic at the tested concentration relative to PEI. The experiment was repeated four times in triplicate. (Dex: dextran; DT: dextran thiol; BA: Butyl amine; HA: hexyl amine; OA: octyl amine; ODA: Octadecyl amine or stearyl amine; DDA: dodecyl amine).
(0.07 MB TIF)

Figure S2 The effect of nanoparticles and EGFP siRNA loaded nanoparticles on BHK-EGFP cells was analyzed. Neither dextran nanoparticles nor EGFP siRNA loaded nanoparticles were cytotoxic at a dose utilized in this study. Growth inhibition was assessed by MTT assay. The experiment was repeated four times in triplicate.
(0.06 MB TIF)
