# Supplementary figures and images for: Inhibition of ABCB1 (MDR1) Expression by an siRNA Nanoparticulate Delivery System to Overcome Drug Resistance in Osteosarcoma
Source: PLoS One. 2010 May 24;5(5):e10764. doi: 10.1371/journal.pone.0010764 (PMC2875382; doi:10.1371/journal.pone.0010764)

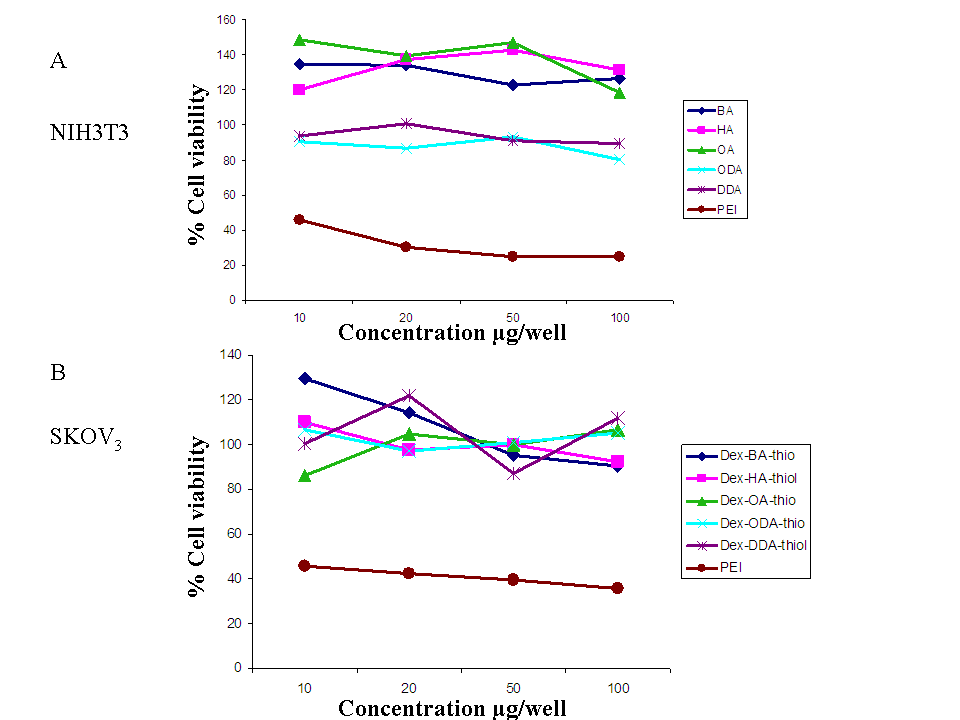

Supplement: Figure S1 — Cytotoxicity of dextran derivatives vs polyethyleneimine(PEI) on NIH3T3 fibroblast (A) and SKOV3 ovarican cancer (B) cells. Varying concentrations of each nanoparticles were added and cells were cultured for 5 days. The mixture of dextran derivatives (thiol+lipid) was found to be almost non-toxic at the tested concentration relative to PEI. The experiment was repeated four times in triplicate. (Dex: dextran; DT: dextran thiol; BA: Butyl amine; HA: hexyl amine; OA: octyl amine; ODA: Octadecyl amine or stearyl amine; DDA: dodecyl amine). (0.07 MB TIF) [file pone.0010764.s001.tif]

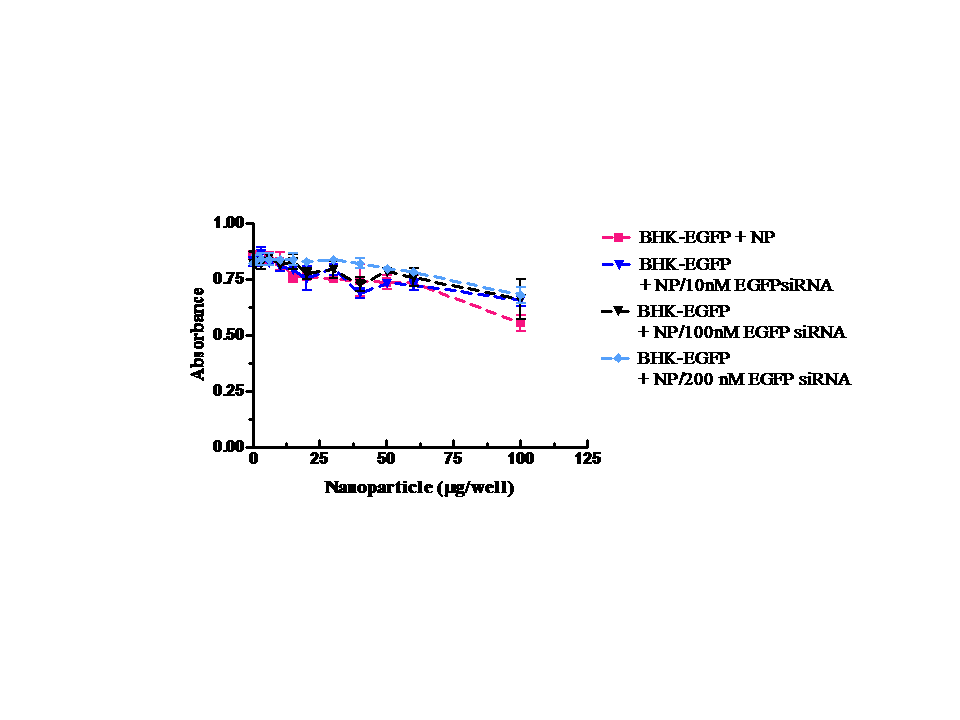

Supplement: Figure S2 — The effect of nanoparticles and EGFP siRNA loaded nanoparticles on BHK-EGFP cells was analyzed. Neither dextran nanoparticles nor EGFP siRNA loaded nanoparticles were cytotoxic at a dose utilized in this study. Growth inhibition was assessed by MTT assay. The experiment was repeated four times in triplicate. (0.06 MB TIF) [file pone.0010764.s002.tif]
